# Salient Region Guided Blind Image Sharpness Assessment

**DOI:** 10.3390/s21123963

**Published:** 2021-06-08

**Authors:** Siqi Liu, Shaode Yu, Yanming Zhao, Zhulin Tao, Hang Yu, Libiao Jin

**Affiliations:** 1 Key Laboratory of Convergent Media and Intelligent Technology (Communication University of China), Ministry of Education, Beijing 100024, China; liu47@cuc.edu.cn (S.L.); yushaodecuc@cuc.edu.cn (S.Y.); yanmingzhao@cuc.edu.cn (Y.Z.); taozl@cuc.edu.cn (Z.T.); 2 School of Information and Communication Engineering, Communication University of China, Beijing 100024, China; 3 School of Aerospace Science and Technology, Xidian University, Xi’an 710126, China; hyu@xidian.edu.cn

**Keywords:** saliency detection, image sharpness, image quality, Gaussian blurring, human vision system

## Abstract

Salient regions provide important cues for scene understanding to the human vision system. However, whether the detected salient regions are helpful in image blur estimation is unknown. In this study, a salient region guided blind image sharpness assessment (BISA) framework is proposed, and the effect of the detected salient regions on the BISA performance is investigated. Specifically, three salient region detection (SRD) methods and ten BISA models are jointly explored, during which the output saliency maps from SRD methods are re-organized as the input of BISA models. Consequently, the change in BISA metric values can be quantified and then directly related to the difference in BISA model inputs. Finally, experiments are conducted on three Gaussian blurring image databases, and the BISA prediction performance is evaluated. The comparison results indicate that salient region input can help achieve a close and sometimes superior performance to a BISA model over the whole image input. When using the center region input as the baseline, the detected salient regions from the saliency optimization from robust background detection (SORBD) method lead to consistently better score prediction, regardless of the BISA model. Based on the proposed hybrid framework, this study reveals that saliency detection benefits image blur estimation, while how to properly incorporate SRD methods and BISA models to improve the score prediction will be explored in our future work.

## 1. Introduction

Human vision system (HVS) is verified as sensitive to the most conspicuous regions in a visual scene, and selective attention is paid to those regions of interest [1,2]. At each moment, the human brain needs to tackle massive messages. Since the amount of information sensed is too high to be completely processed, the brain prioritizes salient regions as the most important cues for follow-up analysis [1]. Emperical and computational studies have reported evidence of a saliency map formed in cortical brain areas or before the primary visual cortex [3,4,5], and this map is used to guide human visual attention to the most relevant regions. This finding inspires increasing applications in object segmentation and pattern analysis for scene understanding [6,7,8].

To identify the most informative and useful regions or objects in an image, salient region detection (SRD) is becoming an increasingly hot topic in the field of computer vision, and many SRD methods have been proposed in the last two decades [9,10,11]. According to the backbone techniques, these SRD methods could be grouped into classic methods and deep-learning-based methods [11], and the former can be further divided into intrinsic- and extrinsic-cue-based methods in terms of the exploited attributes. Regarding the intrinsic cues, an input image is explored to highlight the potential target regions through different aggregation or optimization algorithms in multi-scale or hierarchical analysis. To enhance the feasibility and suppress distractors, various hypotheses are additionally integrated, such as center prior, color prior, objectness prior, global contrast, local contrast and center-surround contrast [12,13,14]. For instance, the most influential method comes from the biologically inspired “center-surround difference” operation, which estimates saliency maps via multi-scale analysis of image color, intensity and orientation [15]. Using a similar framework, a “center-surround divergence” method is designed in an information-theoretic way, and two distributions of visual feature occurrence, derived from a center region and its surrounding region, are compared and finally fused into a single saliency map [16]. In accordance with the understanding of HVS behavior, a center–surround interaction is developed by combining early visual feature extraction, contrast sensitivity analysis, perception decomposition and visual masking, followed by perceptual grouping to bind visual features into a meaningful and high-level structure for saliency detection [17]. However, intrinsic cues might be insufficient, and the resultant performance unsatisfactory, and, thus, extrinsic cues are incorporated, such as ground truth annotation, 3D depth maps and saliency co-occurrence, to overcome the challenges in saliency map estimation [18,19,20]. In [21], the normalized depth prior and the global-context surface orientation prior are proposed, and both priors are integrated with several intrinsic cues for the RGB-D data analysis. In [22], saliency cues are extracted from color images and corresponding depth maps, and, in particular, the feature contrast is derived from color contrast and depth contrast, and the spatial bias is extended from the center bias, both of which are combined for saliency localization and segmentation.

It is unsurpising that deep learning has updated the state-of-the-art in many fields, including, but not limited to, computer vision, precision medicine, and remote sensing [10,23,24,25,26,27]. Since 2015, deep-learning-based methods have also been applied in SRD and achieved promising results [9,10,11]. As one of the most popular tools, deep learning has revolutionized image representation through multi-level and multi-scale abstraction. The deep-learning-based SRD methods can be further categorized from various perspectives, such as backbone network archhitectures, the level of supervision, and learning paradigm [10]. Li and Yu propose a hybrid framework that integrates handcrafted and deep features for saliency detection, and the backbone network is a multi-scale convolutional network, followed by a conditional random field to fuse multiple saliency maps [28]. Based on the maximization of a posteriori principle, Zhang et al. design a novel subset optimization framework to generate a compact set of detection windows of noisy saliency maps, and the proposal of noisy maps is generated by a convolutional neural network (CNN) [29]. For the streamless approximation of saliency maps, fully convolutional networks (FCNs) are used, which perform end-to-end salient object detection. Hu et al. developed a network architecture to learn a level set function to refine the boundaries of the saliency map, and an additional superpixel-guided filter is extended for a more compact saliency output [30]. Besides CNN and FCN, other deep learning architectures, such as an autoencoder, are applied [31,32].

Automatic localization of the most visually relevant areas is useful in many applications, while it is unknown whether salient regions could improve blind image sharpness assessment (BISA). BISA is a distortion-specific task for image quality scoring and requires no reference images [33]. Among the various kinds of distortion, image blurring is more common in daily life. It might be derived from out-of-focus imaging and relative target motion, which degrades image quality and causes difficulties in scene understanding. Since image blur is frequently accompanied by changes in the edges in the spatial domain, and the according high-frequency attenuation, studies are looking into analysis of the edges, contours and image structures. Marziliano et al. compute the spread of edges in the spatial domain and measure the mean edge width (MEW) as a sharpness estimator [34]. Karam et al. first propose the concept of just-noticeable blur via the measure of local contrast in an image, then the concept is incorporated into a probability summation model to score the blurness of the image [35], and later into the cumulative probability of blur detection (CPBD) [36]. Chandler et al. quantify the attenuation of high-frequency messages in the spectral domain via the magnitude spectrum slop and the impact of the contrast component in the spatial domain via the total variation, and propose the spectral and spatial sharpness (S3) indicator [37]. Later, they weight the log-energies of the wavelet sub-band after discrete wavelet transform and design the fast image sharpness (FISH) estimator [38]. Sang et al. estimate the image blurring through the shape of the singular value curve (SVC), since singular values are decreased due to the extent of image blurring [39]. It is also observed that image blur disrupts the structue of local phase coherence (LPC), and then Hassen et al. implement LPC as a sharpness index [40]. Bahrami and Kot consider the maximum local variation (MLV) of each pixel in a small image patch for image blur measure [41]. Gu et al. advance a novel autoregressive-based image sharpness metric by analyzing the autoregressive parameter space of images [42]. Based on an over-complete dictionary of edge patterns, trained with high-quality natural images, Li and his colleagues propose a sparse representation based image sharpness (SPARISH) metric [43]. They also represent a noticeable blur with the magnitudes of discrete orthogonal moments, and design the blind image blur evaluation (BIBLE) model [44]. Furthermore, they collect a total of eleven sharpness-aware features in multi-scale spatial and spectral domains, and the robust image sharpness evaluation (RISE) model is formulated [45]. Sun et al. simulate the impact of viewing distance on blur distortion, and calculate the distribution characteristics of the local maximum gradient of multi-resolution images in the spatial domain [46]. Cai et al. design an interesting procedure that reblurs the orignial blurred image [47], and the global sharpness is estimated through inter-resolution self-similarities, since the discrepancy between an image and its reblurred version indicates the extent of blur in the image. CNN has also been used for BISA tasks. Limited by a small number of training samples, Yu et al. propose a hybrid framework to incorporate a shallow CNN for representation learning of image sharpness, and the prediction performance is further boosted by different kinds of numerical regressors [25,48]. Their further kernel visualization indicates that image sharpness is related to image edges and structures [25]. Li et al. design semantic feature aggregation for blur-dominated distortions, which alleviates the impact of image content variation, and the results suggest that deep semantic features might address image content variation in image-quality assessment [49]. In addition, Hosseini et al. synthesize the HVS response as a linear combination of finite impulse response derivative filters, and the falloff of high band frequency magnitudes is stimulated in natural imaging paradigm [50].

Several studies have used saliency detection for image quality estimation. In [51], the visual saliency map is set as a weighting function with the purpose of improving the existing image quality metrics. In [52], an image is divided into regions of interest and background regions, and computational metrics from these regions are pooled to score the image quality. In [53], visual saliency is usede to compute the local quality map and then as a weighting function to reflect the importance of a local region for final quality assessment. In [54], visual saliency information is introduced into image quality metrics, and the correlation performance can be further boosted between the predicted scores and the subjective scores. In [55], image quality degradation is modelled as saliency deviation, and both the saliency map and quality information are embedded into the proposed metric. In these studies [51,52,53,54,55], saliency maps are used as a weighting function, quantitative features, or degradation results in an implicit manner to improve the prediction of image quality.

However, the effect of salient regions on image quality assessment is still unknown. To better analyze this effect, this study concerns a specific distortion of Gaussian blurring, and it excludes the effect of other kinds of distortion variations. A hybrid salient-region-guided BISA framework is designed. This reorganizes the output of an SRD method as the input of a BISA model and, thus, the change in the BISA metric value can be directly related to the change in the BISA input. In summary, the contributions of this study are as follows:

1. A salient region guided BISA framework is designed. It aims to observe the effect of salient region input on image blur estimation;
2. Based on the proposed framework, the effect of SRD results on the BISA prediction is investigated. Specifically, this involves three SRD methods combined with 10 BISA models, and the experiment is conducted on three Gaussian blurring image datasets;
3. Experimental results reveal that salient regions can help a BISA model achieve a comparable performance to one using whole-image input. In particular, the saliency optimization from robust background detection (SORBD) method is suggested.

The remainder of this paper is organized as follows. The details of the involved datasets, SRD methods, BISA models, and experiment design are presented in Section 2. Experimental results are reported in Section 3. The findings and limitations of this study are summarized in Section 5. Finally, the conclusions are included in Section 5.

## 2. Materials and Methods

### 2.1. Data Collection

Synthesized Gaussian blurring images are collected from three widely used datasets. CSIQ [56] and LIVE [57] contain 30 and 29 pristine images, respectively. In these datasets, images are distorted by five different levels of blurring, and image quality is reported using differential mean opinion scores. The third dataset TID13 [58] has 25 reference images. Each image in the database is degraded by four different levels of Gaussian blurring, and image quality is reported using mean opinion scores.

**Figure 1:**
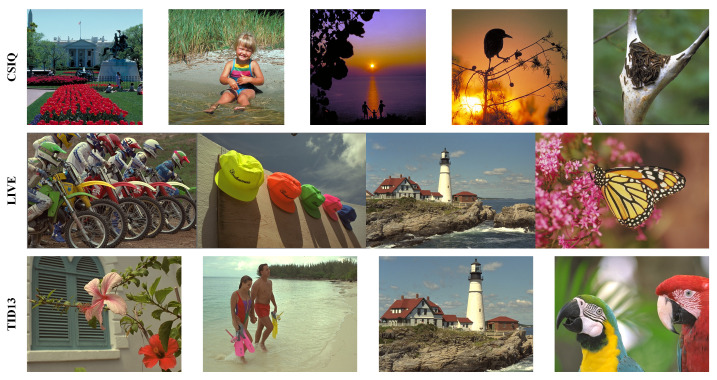
Several representative pristine images in the three datasets.

The pristine images are from a set of source images and reflect adequate diversity in terms of image content, including pictures of faces, people, animals, close-up shots, wide-angle shots, nature scenes, man-made objects, images with distinct foreground/background configurations, and images without any specific object of interest. Figure 1 demonstrates several representative images in these datasets. Interestingly, it was found that the refernce images in CSIQ are distinctive from those in the other two databases, while more than ten images have the same or similar content as that shared in LIVE and TID13; for instance, the lighthouse, as shown in Figure 1.

In the three databases, Gaussian blurring is synthesized intentionally. To each pristine image, its R, G, and B channels are filtered using a circular-symmetric 2-D Gaussian kernel of standard deviation (δ) of Gaussian distribution [57], as shown in Equation (1), where *c* is a color channel, δ stands for the standard deviation of the distribution, *x* and *y* are the location indices of pixels. The three color components of an image are blurred using a same kernel, while, to different images, the values of δ change in different ranges.

(1) $$G_{2D}^{c}\left(x,y,\delta \right)=\frac{1}{2\pi \delta^{2}}\exp\left(-\frac{x^{2}+y^{2}}{2\delta^{2}}\right),$$

### 2.2. Evaluated SRD Methods

Three SRD methods are evaluated. One detects salient regions with diffusion process on a 2-layer sparse graph (DPLSG) [59]. It constructs the diffusion matrix using sparse graph and obtains the seed vector from the spatial variance of superpixel clusters. DPLSG computes two coarse saliency maps via the foreground and the background seed vectors, and the final saliency map is generated using a manifold ranking diffusion method. The second method, the saliency optimization from robust background detection (SORBD) [60], designs a robust background measure to compute boundary connectivity, which characterizes the spatial layout of image regions. Moreover, it integrates multiple low-level cues into the optimization framework with an intuitive geometrical interpretation. The last method incorporates both salient region localization and image segmentation (SRIS) into a hybrid framework and aims to overcome image noise and artifacts [61]. It uses an adaptive level-set evolution protocol and an adaptive weight function is embedded. SRIS can balance both the internal and the external functions and tackle different kinds of image distortion, such as inhomogeneity and noise.

The reasons for using the three SRD methods are manifold. Firstly, the three methods consider saliency detection from various perspectives. DPLSG considers sparse graph and manifold ranking diffusion [59], SORBD computes the boundary connectivity and integrates low-level geometric cues for optimization [60], and SRIS designs an adaptive level-set evolutional protocol and an adaptive weight function to tackle different kinds of image distortion [61]. Secondly, the SRD methods are able to estimate the saliency maps of the Gaussian blurring images. Offline experiments were also conducted on two deep-learning-based SRD methods (BASNet [62] and PiCANet [63]). Since image contextures or boundaries are important, both methods cause failure cases on highly blurry images. Last, but not least, the codes of the three SRD methods are avaialable online, and the effort involved in algorithm implementation is relieved.

### 2.3. Involved BISA Models

This study involves 10 BISA models, including MEW [34], CPBD [36], S3 [37], FISH [38], SVC [39], LPC [40], MLV [41], SPARISH [43], BIBLE [44], and RISE [45]. The methods are mainly implemented with MATLAB. Codes are available online and the methods are tested without any modifications.

Notably, deep-learning-based BISA models were also explored [25,49]. Deep learning requires a large number of samples for model training. To tackle this problem, Yu et al. [25] randomly select hundreds of patches from an image, and the score of each patch is paired with that of the image. Li et al. [49] represent an image by using multiple overlapping patches with the purpose of avoiding introducing unwanted geometric deformation and retaining the model performance. However, the sizes of salient regions are decreased, and some key parameters in deep learning-based models become meaningless, such as the patch number [25] and the stride [49]. In other words, the deep-learning-based BISA models [25,49] cannot be well trained and, consequently, the effect of salient regions on deep-learning-based BISA performance becomes ambiguous. Therefore, deep-learning-based BISA models are not considered in this study.

### 2.4. The Proposed Hybrid Framework

**Figure 2:**
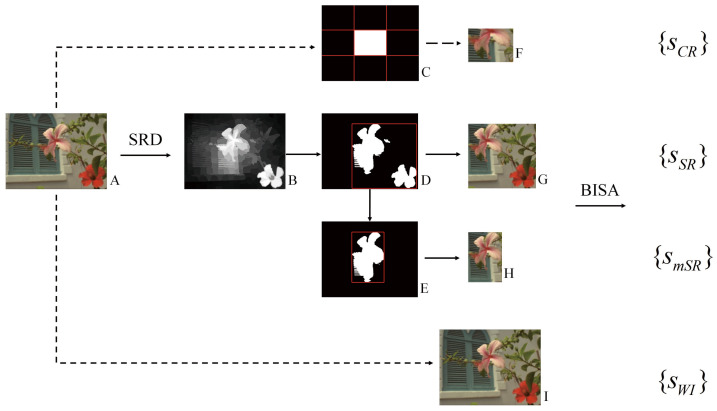
The proposed hybrid framework and experiment design. Under different image input scenarios, the performance of salient-region-guided image sharpness estimation is compared.

The proposed hybrid framework is shown in Figure 2. It uses SRD methods to generate saliency maps to guide BISA tasks (the arrows with solid line). An SRD method computes the saliency map (B) of an input image (A). Then, the saliency map is binarized into foreground masks, and a minimum outer rectangle is generated accordingly (D). Next, two kinds of salienct-region-guided BISA experiment are conducted. One takes the image region in the rectangle as the input (G), and the other uses the binary mask with the largest area (E) to generate its outer rectangle and to obtain the image region as the input (H). For fair comparison, two additional experiments are carried out. One uses the source image as the input (I), and the other takes the center area (C) of the image as the input (F), since the center region, as a center prior, assumes salient objects are more likely to be located at the center of an image [11]. Therefore, to each BISA model, four kinds of experiments are conducted by using different image inputs (F-I). Finally, by using different kinds of image input, including center region (CR), salient region (SR), major salient region (mSR) and whole image (WI), a BISA model predicts the scores as $\left\{s_{CR}\right\}$, $\left\{s_{SR}\right\}$, $\left\{s_{mSR}\right\}$ and $\left\{s_{WI}\right\}$, respectively.

### 2.5. Performance Evaluation

Two metrics, Pearson linear correlation coefficient (PLCC) and Spearman rank-order correlation coefficient (SRCC), are used to evaluate the performance of BISA models. The former measures the prediction accuracy, and the latter assesses the prediction monotonicity. The values of both metrics range between 0 and 1, and a higher value indicates a better prediction.

Beefore quantifying the prediction performance, a nonlinear fitting is routinely used to map the predicted scores to the range of corresponding subjective ratings. There are two fitting methods. Since little difference is found [64], this study uses the five-parameter-based curve fitting method, shown below,

(2) $$Q\left(s\right)=q_{1}\left(\frac{1}{2}-\frac{1}{1+\exp q_{2}\left(s-q_{3}\right)}\right)+q_{4}s+q_{5},$$

where *s* and Q(s) correpond to the input scores (such as $\left\{s_{CR}\right\}$) and the mapped scores, and $q_{i}$ (i=1,2,⋯,5) are determined during the score fitting.

### 2.6. Experiment Design

The experiment design is described below:

1. Experiments are conducted on different combinations of SRD methods and BISA models, and the prediction performance is illustrated;
2. On each dataset, the BISA model with the largest score drop between the WI input and the CR input is investigated. The procedure consists of three steps. First, compute the difference in PLCC values derived from the WI and the CR input, and the BISA model with the largest score drop is determined. Then, to the BISA model, absolute difference of prediction scores between the WI input and the CR input is shown, and the images causing the three largest score drop are determined. In the end, the WI, the CR, the SR, and the mSR are also demonstrated;
3. The results from the CR input are set as the baseline, and the difference obtained by subtracting the value (PLCC and SRCC) of the CR input from that of the corresponding SR and mSR input is shown;
4. The size of salient regions (SR and mSR) is analyzed and illustrated to verify the effectiveness of SRD methods;
5. The average running time of SRD methods and BISA models is reported separately.

### 2.7. Software and Platform

Software runs on a Windows system. The system is embedded with Intel(R) Core(TM) i7-8700 CPU (3.20 GHz), 16 GB RAM and one GPU card (Nvidia GeForce GTX 1070, Nvidia, Santa Clara, California, United States). The SORBD method is implemented by Python 3.6, and other codes of SRD methods and BISA models are implemented by Matlab R2018a. It is worth noting that some codes need executable files to run. For a fair comparison, these algorithm codes are accessible on GitHub (https://github.com/NicoYuCN/srgBISA (accessed on 5 June 2021)).

## 3. Results

**Figure 3:**
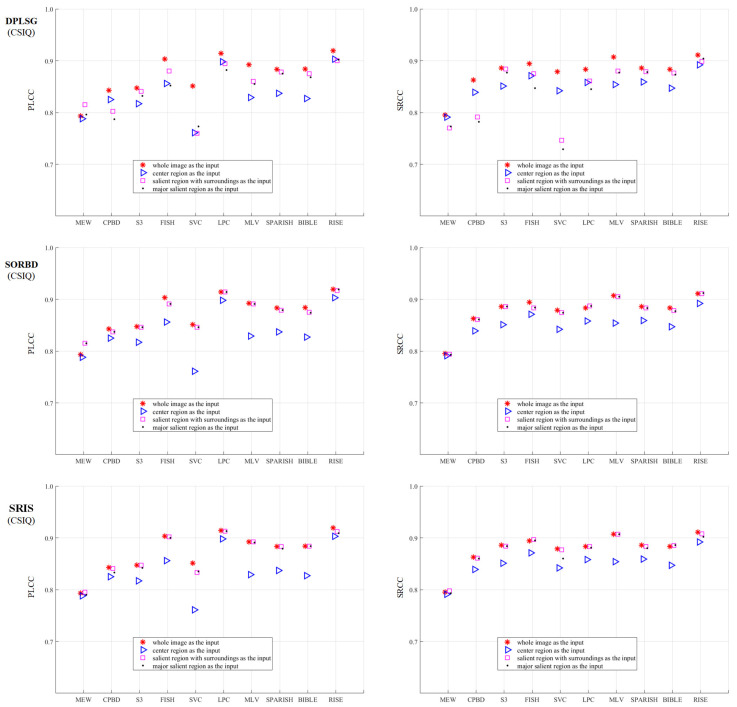
Comparison of prediction scores regarding different inputs on the CSIQ Gaussian blurring images. This indicates that using SR (pink square □) or the mSR (black dot ·) as input could achieve close results to the corresponding WI input (red star ∗), and their results are better than that using the CR (blue triangle Δ) input. (The figure can be enlarged for viewing).

**Figure 4:**
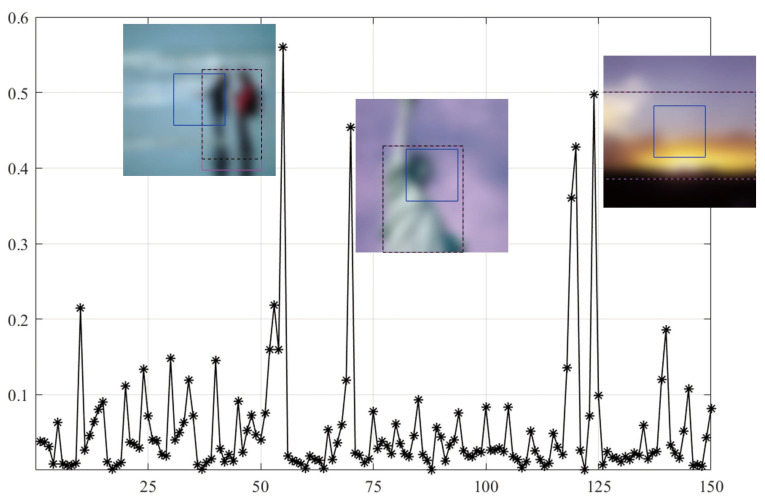
Absolute difference in predicted scores on CSIQ images based on the SORBD method combined with the SVC model. The images causing the three largest score drops are illustrated next to the points. (The figure can be enlarged for viewing).

**Figure 5:**
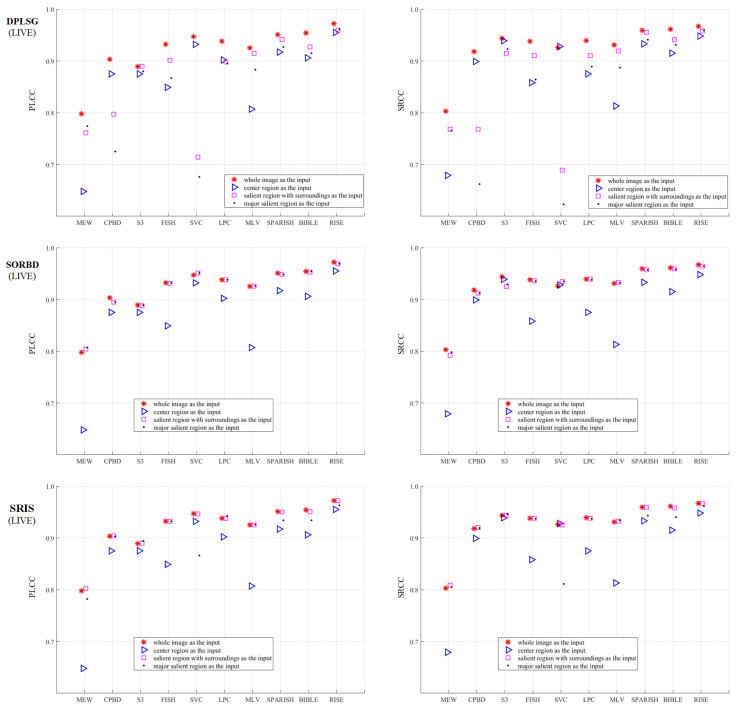
Comparison of BSIA results with different inputs from SRD methods on the LIVE Gaussian blurring images. This shows that the SR (□) or the mSR (·) input could achieve comparable results to the WI input (∗), and the CR (Δ) input leads to the worst prediction performance. (The figure can be enlarged for viewing).

**Figure 6:**
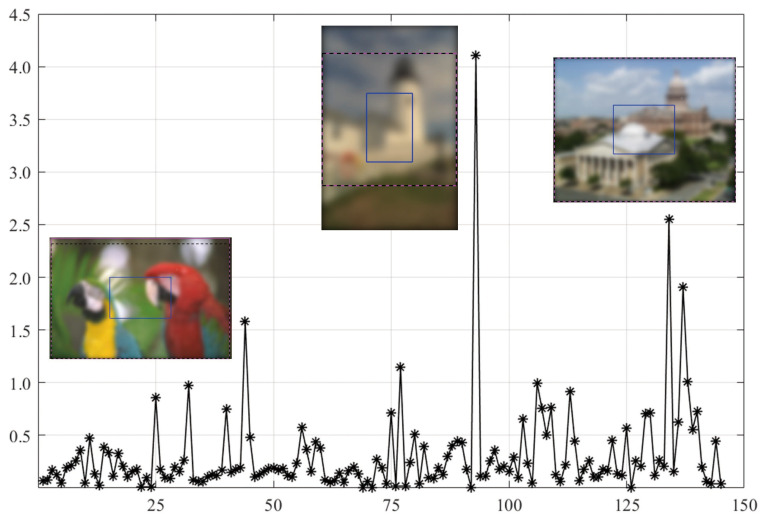
Absolute score difference in each image in LIVE based on the SORBD method and the MEW model. Beside the points of the three largest score drops, the corresponding images are illustrated. (The figure can be enlarged for viewing).

**Figure 7:**
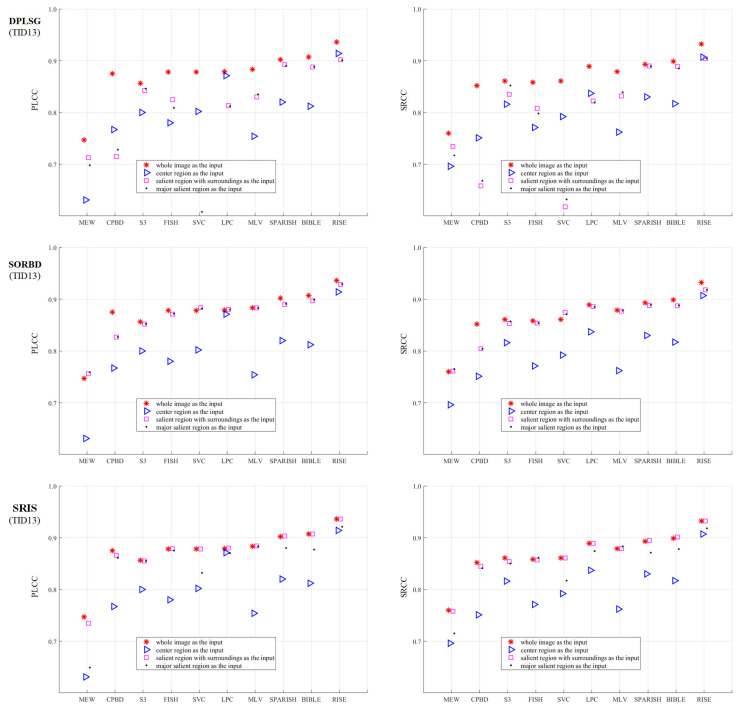
Comparison of BSIA performance on the TID13 Gaussian blurring images. The metric values using salient region inputs are slightly inferior to those obtained from the WI input (∗), while the values are much better than those obtained using the CR input (Δ). (The figure can be enlarged for view).

**Figure 8:**
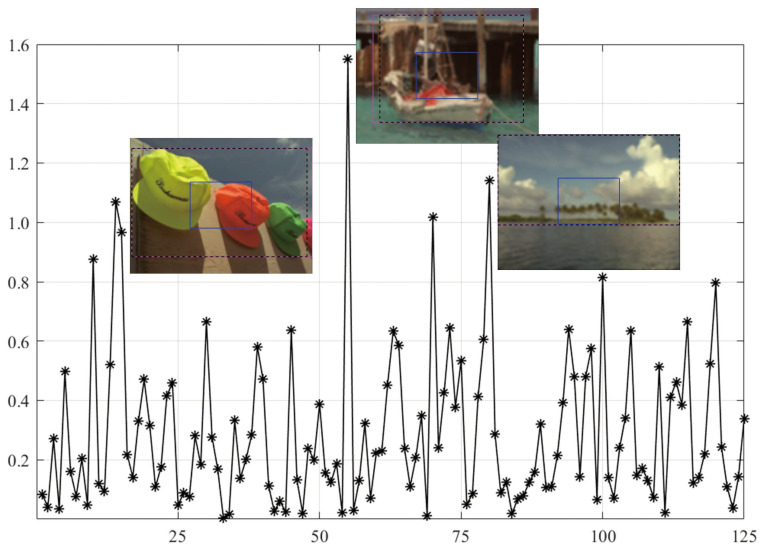
Absolute score difference in each image in TID13 using the SORBD method and the MEW model. The corresponding images are given next to the points of the three largest score drops. The three images show various levels of blurring distortion. (The figure can be enlarged for view).

Based on the proposed framework, the effect of salient region inputs on the BISA performance is investigated. A total of three SRD methods combined with ten BISA models are explored, and three datasets (CSIQ, LIVE, and TID13) are involved in the quality assessment of Gaussian blurring images. The prediction performance is quantified with PLCC and SRCC values. To each dataset, the results are demonstrated from three parts:

1. The metric values are shown in Figures 3, 5 and 7. To differentiate the inputs, WI (red star ∗), CR (blue triangle Δ), SR (pink square □); and mSR (black dot ·) are denoted with different combinations of colors and markers.
2. The BISA model with the largest score drop between the WI and its CR input is shown in Figures 4, 6 and 8. The reasons why the BISA model causes the lowest PLCC value are also provided. Note that SORBD is used as the default SRD method;
3. Setting the performance of the CR input as the baseline, the difference obtained by subtracting the baseline from that of using SR and mSR inputs is shown in Table 1, Table 2 and Table 3. Accordingly, if the BISA metric value of a salient region input is smaller than the baseline performance, its sharpness metric value is negative.

**Figure 9:**
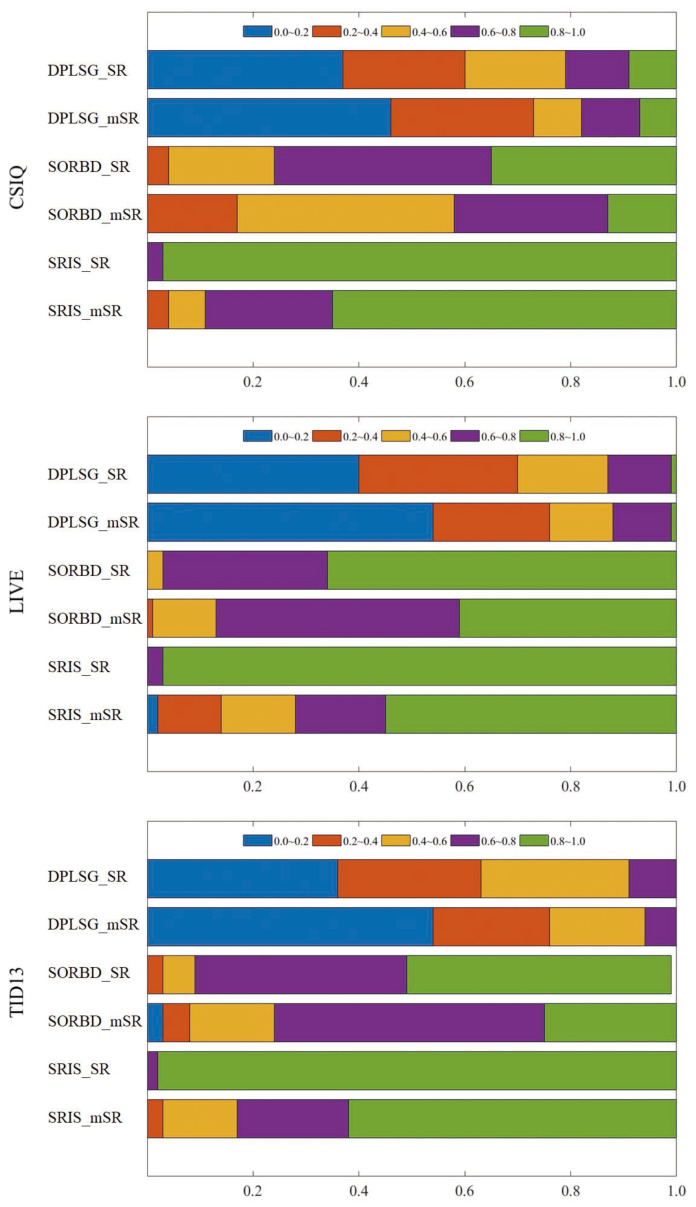
Distribution of the size proportion of used salient regions over the corresponding whole images. The size proportions are divived into five bins and denoted with different colors. (The figure can be enlarged for viewing).

Besides the three parts, the distribution of size proportions of SR and mSR input is shown in Figure 9 and Table 4, and the running time is listed in Table 5 and Table 6. In the end, the SRD method with good balance between computing efficiency and prediction performance is suggested.

### 3.1. On the CSIQ Gaussian Blurring Images

Figure 3 shows the BISA results on the CSIQ Gaussian blurring images. The SR input (□) leads to equal or comparable metric values to the WI input (∗), followed by the mSR input (·), and the CR input (Δ) obtains the worst BISA results. In general, SORBD and SRIS lead to slightly higher prediction scores than DPLSG for the same BISA model (except the MEW). Based on SORBD or SRIS, several BISA models, such as MEW, CPBD, S3, LPC, MLV, SPARISH and BIBLE, obtain reliable scores when the WI is replaced by SR or mSR, but not the CR input. Interestingly, among the BISA models, MEW with salient regions from DPLSG or SORBD achieves superior PLCC values in comparison to that using the WI input.

As shown in Figure 3, SVC generally achieves low PLCC scores compared to the BISA models. Subsequently, based on the SORBD method, followed by the SVC model, the absolute score difference in each image is shown in Figure 4, and the images are illustrated, besides the points of the three largest score differences. In the figure, the horizontal axis shows the image index and the vertical axis shows the score difference. It can be seen that the three images are highly blurry and the CR covers only a part of the salient region. In contrast, using the salient regions from SORBD as an input leads to a small score drop (Figure 3), because the detected salient regions retains most of edge structures and image content.

Moreover, in comparison with the BISA results, by using the CR input, the performance difference is quantified and shown in Table 1. Note that a negative metric value denoted using the salient region input results in a worse prediction performance than that using the CR input. Overall, both the SR and the mSR input could enhance the metric values. Among the SRD methods, SORBD enables the PLCC values of from 0.012 to 0.085 higher, and the SRCC values of from 0.003 to 0.051 higher. Among the BISA models, S3, MLV, SPARISH and BIBLE obtain a consistently positive performance. For instance, MLV obtains an increase of PLCC values in the range between 0.026 and 0.063 and SRCC values in the range between 0.026 and 0.054.

### 3.2. On the LIVE Gaussian Blurring Images

As shown in Figure 5, the performance of BISA models combined with SRD methods is evaluated on the LIVE Gaussian blurring images. This shows that the SR input (□) obtains competitive metric values compared with the WI input (∗), followed by the mSR (·) and the CR input (Δ). Among the SRD methods, when using the SR and mSR input, the DPLSG, when combined with MEW, CPBD, FISH, SVC or MLV models, causes an obvious metric decrease, and SORBD and SRIS lead to a slightly inferior performance for each BISA model. Notably, using SORBD or SRIS, most BISA models, including CPBD, S3, FISH, SVC, LPC, MLV, SPARISH, BIBLE and RISE, maintain the prediction scores well. In addition, the salient region detected by SORBD and then combined with MEW or SVC can improve the PLCC values, more than the WI input for image blur estimation.

As shown in Figure 5, the MEW model obtains the lowest PLCC score when using the CR input. Based on the SORBD method combined with the MEW model, the absolute score difference in each image is shown in Figure 6, where the horizontal axis shows the image index, and the vertical axis shows the score difference. In addition, images leading to the three largest score drops are illustrated. The figure shows that the images are blurry, and the center regions cover relatively few edge structures in comparison to the whole images, both of which are important to the MEW model in image blur estimation. In contrast, when using salient regions as input, the score drop is relatively small, because the regions retain most edge structures and scene content.

Moreover, Table 2 shows the BISA difference between the CR input and the salient region input. Note that a negative metric value denotes that the salient region input had a worse prediction performance than the CR input. In general, both the SR and the mSR input enhance the metric values. On the LIVE Gaussian blurring images, 6 out of 10 BISA models consistently improve the values of evaluation metrics. Notably, the MEW model increases the PLCC value and the SRCC value up to 0.159 and 0.130 higher, respectively.

### 3.3. On the TID13 Gaussian Blurring Images

The metric values obtained using BISA models combined with SRD methods are shown in Figure 7. Compared to the WI input (∗), the SR (□) maintains the BISA performance well, followed by the mSR input (·), both of which outperform the CR input (Δ). Notably, SORBD combined with BISA models (except the CPBD model), and SRIS with CPBD, S3, FISH or MLV, achieve competitive BISA results compared to the corresponding WI input, while DPLSG leads to an observable decrease in BISA scores. In addition, based on the salient regions detected by SORBD, the BISA models, such as MEW and SVC, achieve even higher metric values than the WI input.

As shown in Figure 7, the MEW model obtains the lowest PLCC score when using the CR input. Based on the SORBD method combined with the MEW model, the absolute score difference in each image is shown in Figure 8 where the horizontal axis shows the image index and the vertical axis shows the score difference. In addition, the images causing the three largest score drops are illustrated. This indicates the three images have various levels of blurring distortion. Moreover, the CR input covers limited edge structures, while the detected salient regions retain most or all of the edge structures.

Based on the TID13 Gaussian blurring images, Table 3 shows the results of a comparison between salient region inputs and the CR input from PLCC and SRCC values. By setting the CR input as the baseline, the quantitative difference indicates that most BISA models achieve a better performance. Specifically, the MEW model has a 0.128 higher PLCC value and a 0.069 higher SRCC value, and the MLV model obtains an even better improvement. Regarding the SRD methods, SORBD and SRIS outperform the DPLSG method.

### 3.4. Distribution of the Size Proportion of Salient Regions

The distribution of the size proportion of used salient regions over all the images is shown in Figure 9. The size proportions are divived into five bins with equal intervals, which are highlighted by different colors. On the CSIQ dataset, the size proportion of mSR input generated from the DPLSG, the SORBD and the SRIS mainly falls into the range of (0.0, 0.2], (0.4, 0.6] and (0.8, 1.0], respectively. The similar phenomenon is also observed on the other two datasets.

In addition, the average size proportion of salient regions (SR and mSR) over the whole image is summarized in Table 4. This indicates that the DPLSG method generates a relatively smaller SR and mSR, followed by the SORBD method, while the SRIS method produces an SR input of approximately the same size as the whole image.

### 3.5. Time Consumption

The average time consumption per image with regard to different SRD methods is shown in Table 5. It was found that the DPLSG method requires more than 3.00 s to compute the saliency map of an image, while SORBD takes less than 1.00 s.

Table 6 summarizes the time cost per input, on average, regarding different BISA models. In general, when using WI input for image blur estimation, S3 and SPARISH are the most time-consuming models, taking more than 7.00 s, RISE and LPC require less than 2.00 s, and the time cost of other models is less than 1.00 s. When using CR, SR and mSR inputs, the time consumption drops, since only a part of the image region is used to estimate image sharpness.

## 4. Discussion

This study proposed a hybrid framework for no-reference image blur estimation. Based on the framework, the effect of salient region input on BISA tasks was investigated. It involved three SRD methods and ten BISA models. The procedure was conducted on three Gaussian blurring image datasets. Specifically, a BISA model’s input comes from different parts of an image, including WI, CR, SR and mSR.

The BISA performance (PLCC and SRCC) based on salient regions is comparable to that using the WI input (Figure 3, Figure 5, and Figure 7). For instance, on the LIVE (Figure 5), the SORBD method enables the BISA models, such as MEW, CPBD, S3, LPC, MLV, SPARISH and RISE, retain the estimation of image quality well. Moreover, SORBD jointly with MEW, leads to even higher PLCC values compared to the WI input. A similar phenomenon is observed on the other two image datasets (Figure 3 and Figure 7). This indicates that the performance using detected salient regions could approximate that using whole images.

However, quite a bit of variation in the metric values is observed for each BISA model. This shows that the PLCC value of SVC is generally low in CSIQ (Figure 3), and the PLCC and SRCC values of MEW are generally low in LIVE (Figure 5) and TID13 (Figure 7). FTo further understand this, we used the SORBD method for saliency detection to found out why the SVC model on CSIQ and the MEW model on LIVE and on TID13 lead to inferior performance. Correspondingly, the absolute score difference on each image and the images causing the three largest score drops are illustrated in Figure 4, Figure 6 and Figure 8. The reasons for the variations in metric values are manifold. From the perspective of datasets, the images in CSIQ are distinct from those in the other two datasets, while more than ten images have the same or similar content in LIVE and TID13, such as the reference images, named lighthouse, parrots, bikes, sailing, woman, and statue in the LIVE dataset. Thus, the discovery of variation in metric values is meaningful. Secondly, from the principle of the BISA models, SVC focuses on singular value analysis, and MEW estimates the edge width. As shown in Figure 4, the center region of images is homogeneous because of limited contrast. Thus, the curve in singular values struggles to reflect the curve in the whole image, while the mean edge width of the center region might be close to that of the whole image. On the other hand, as shown in Figure 6 and Figure 8, the center regions are inhomogeneous. The curve in singular values might be close to the curve in the whole image, while a gap in edge-width values might exist between the center region and the salient regions. In addition, the center-region or the salient-region input is quite different from the whole-image input. Thus, the predicted scores are correspondingly changed, which might accumulatively affect the score nonlinear fitting and the BISA metric values.

In comparison to the CR input, corresponding salient regions (SR and mSR) lead to better BISA performance. On the LIVE images (Table 2), there are six BISA models that consistently increase the metric values. The MEW model dramatically enhances the PLCC and the SRCC up to 0.159 and 0.130 higher, respectively. Similar phenomenon are also observed on the other two databases (Table 1 and Table 3). This suggests that the use of center regions is insufficient, while using salient regions seems more appropriate in image blur estimation. Accoding to the review of SRD methods [11], center bias is the most popular bias, and important objects are composed in the center of an image. This bias is widely used in SRD, but is rare in image-quality estimation. Layek et al. [66] propose an image quality metric that uses visual saliency and contrast, and extra attention is paid to the center by increasing the sensitivity of the similarity maps between the reference image and the distorted image. In their study, an image is splitted into 3 × 3 blocks, as shown in Figure 2D, and, to enhance the prediction performance, not only the center region but also its whole image are quantified from saliency similarity and contrast similiarty. Thus, the center region is not adequate to represent the whole image in image quality estimation. In other words, center bias or center-surround priors could help in silency map localization, while, in image quality estimation, salient regions are more informative and helpful.

The difference in prediction performance is observed among BISA models combined with SRD methods. In general, the detected salient regions from the SORBD and SRIS methods are suitable for the MEW, FISH, LPC, MLV, SPARISH and BIBLE models, and the regions from the DPLSG method are helpful for the models of SPARISH and BIBLE, in the utimate goal of image blur estimation. Among the models, MEW quantifies the spread of edges [34], FISH weights the log-energies of wavelet sub-band [38], LPC analyzes the coherence of local phases [40], MLV measures the maximum local variation in pixels [41], SPARISH concerns image edge patterns from an over-complete dictionary [43], and BIBLE measures the magnitudes of orthogonal moments [44], all of which could benefit from saliecy detection to avoid unreliable edge detection, energy weighting, coherence analysis, variation measure, pattern reconstruction, and magnitue computing, respectively. For saliency detection, DPLSG computes the diffusion matrix and seed vectors are obtained from the spatial variance in superpixels [59], SORBD integrates spatial layout, low-level cues and geometric interpretation for robust background measure [60], and SRIS concerns the effect of image inhomogeneity, noise and artificats, and emphasizes level-set evolution protocol [61]. However, image blur imposes additional difficulties to the diffusion matrix computation [59], and the strategies from both [60,61] might tackle these challenges, while the generailization capacity of these methods on the three datasets needs further quantification.

Most importantly, the distribution proportion of the sizes of used salient regions verifies its practical advantage. That is, saliency detection could decrease the computational burden (Figure 9 and Table 4) and the time cost in BISA tasks (Table 6). Specifically, DPLSG requires only from 30% to 36% of the image region and SORBD needs from 71% to 87% of image region to guide the BISA task. Overall, among the three SRD methods, SORBD is identified as the best one for tradeoff saliency detection, time consumption and blur estimation (Table 5). On the other hand, DPLSG shows superiority when used in combination with several BISA metrics, such as MEW, S3, FISH, and RISE, with a slight decrease in metric values. To our knowledge, several studies [51,52,53,54,55] have verified the added value of visual saliency maps in the estimation of image quality. Suprisingly, Ref. [65] shows that the profit extent seems not to be directly relevant to the performance of human fixation prediction when using the saliency detection method. This might suggest that salient regions are different from human fixation regions. The former reveals the conspicuous regions in a visual scene, while the latter aims to process the scene for increased image understanding. Thus, the incorporation of BISA models and SRD methods should be properly designed.

There are several limitations to the current study. First, most SRD methods take advantage of data biases, prior knowledge and image contrast in the algorithm design [11], and these methods might not be suitable for Gaussian blurring. The study [54] shows that blurred images could be divided into pattern and non-pattern groups, and most salient regions are more influential on pattern images than non-pattern images. Thus, a detailed study is required to verify the capacity of SRD methods on the general natural image test. Second, image blur imposes additional challenges for saliency detection. Future development of saliency-guided BISA models should consider the impact of image blur on saliency map detection in advance. Third, no deep-learning-based SRD methods or BISA models are evaluated. Deep learning requires massive training samples with a fixed image size, while it is difficult for an SRD method to generate salient regions with a consistent output size from images with different content. Specifically, our offline experiments figured out the infeasibility of deep learning methods. In addition, this study concerns only synthesized Gaussian blurring. Other image distortions could be evaluated in the proposed framework and, in the near future, the effect of detected salient regions on image-quality estimation can be fully understood.

## 5. Conclusions

This study proposed a hybrid framework to investigate the effect of detected salient regions on image blur assessment. This involved three saliency detection methods and ten blind image sharpness estimators, and experiments were conducted on three Gaussian blurring image datasets. Experimental results verified that salient regions benefit from image blur estimation and, most importantly, salient regions might be used as the surrogate of whole images for image quality assessment to enhance computing efficiency. The aim of future work will be to improve the prediction performance, develop accurate and generalizable saliency detection methods and properly incorporate SRD methods and BISA models.

## Figures and Tables

**Table 1:** Center region (CR) input as the baseline for BISA comparison on the CSIQ.

			MEW	CPBD	S3	FISH	SVC	LPC	MLV	SPARISH	BIBLE	RISE
SR input	DPLSG	PLCC	0.027	−0.023	0.024	0.024	0.000	−0.004	0.030	0.041	**0.048**	−0.003
		SRCC	−0.021	−0.049	**0.033**	0.005	−0.095	0.003	0.026	0.021	0.029	0.007
	SORBD	PLCC	0.028	0.012	0.029	0.035	**0.085**	0.016	0.062	0.042	0.048	0.014
		SRCC	0.003	0.021	0.034	0.012	0.032	0.029	**0.051**	0.025	0.031	0.019
	SRIS	PLCC	0.007	0.015	0.031	0.046	**0.072**	0.015	0.063	0.046	0.057	0.009
		SRCC	0.007	0.022	0.033	0.027	0.035	0.025	**0.054**	0.024	0.038	0.016
mSR input	DPLSG	PLCC	0.008	−0.039	0.016	−0.004	0.012	−0.016	0.026	0.037	**0.042**	−0.002
		SRCC	−0.017	−0.057	0.025	−0.024	−0.113	−0.013	0.023	0.020	**0.026**	0.012
	SORBD	PLCC	0.027	0.012	0.029	0.035	0.085	0.016	**0.062**	0.042	0.047	0.016
		SRCC	0.003	0.021	0.035	0.013	0.033	0.029	**0.051**	0.025	0.030	0.020
	SRIS	PLCC	0.002	0.007	0.025	0.044	**0.074**	0.015	0.061	0.041	0.057	0.006
		SRCC	0.002	0.021	0.033	0.024	0.018	0.022	**0.054**	0.022	0.039	0.010

**Table 2:** Center region (CR) input as the baseline for BISA comparison on the LIVE.

			MEW	CPBD	S3	FISH	SVC	LPC	MLV	SPARISH	BIBLE	RISE
SR input	DPLSG	PLCC	**0.113**	−0.078	0.014	0.052	−0.219	−0.004	0.109	0.024	0.021	0.005
		SRCC	0.088	−0.130	−0.024	0.052	−0.239	0.035	**0.106**	0.022	0.026	0.011
	SORBD	PLCC	**0.156**	0.019	0.013	0.082	0.019	0.036	0.119	0.031	0.047	0.014
		SRCC	0.113	0.013	−0.014	0.078	0.007	0.064	**0.120**	0.024	0.044	0.016
	SRIS	PLCC	**0.155**	0.029	0.015	0.083	0.013	0.036	0.118	0.032	0.045	0.017
		SRCC	**0.130**	0.021	0.005	0.080	-0.004	0.063	0.118	0.025	0.043	0.019
mSR input	DPLSG	PLCC	**0.125**	−0.151	0.005	0.017	−0.256	−0.006	0.076	0.009	0.009	0.006
		SRCC	**0.086**	−0.237	−0.016	0.006	−0.305	0.014	0.074	0.008	0.016	0.011
	SORBD	PLCC	**0.159**	0.019	0.014	0.082	0.019	0.036	0.119	0.031	0.047	0.014
		SRCC	**0.119**	0.014	−0.010	0.077	0.006	0.063	**0.119**	0.024	0.043	0.017
	SRIS	PLCC	**0.133**	0.027	0.019	0.083	−0.066	0.040	0.120	0.016	0.028	0.008
		SRCC	**0.126**	0.020	0.007	0.079	−0.117	0.062	0.120	0.010	0.024	0.013

**Table 3:** Center region (CR) input as the baseline for BISA comparison of the TID13.

			MEW	CPBD	S3	FISH	SVC	LPC	MLV	SPARISH	BIBLE	RISE
SR input	DPLSG	PLCC	**0.083**	−0.052	0.042	0.045	−0.226	−0.057	0.075	0.072	0.076	−0.013
		SRCC	0.038	−0.093	0.019	0.037	−0.174	−0.015	0.070	0.060	**0.072**	−0.003
	SORBD	PLCC	0.126	0.060	0.052	0.092	0.082	0.010	**0.128**	0.070	0.085	0.014
		SRCC	0.065	0.053	0.037	0.083	0.082	0.049	**0.115**	0.058	0.071	0.011
	SRIS	PLCC	0.105	0.099	0.056	0.099	0.075	0.009	**0.129**	0.083	0.095	0.021
		SRCC	0.062	0.094	0.038	0.086	0.069	0.052	**0.117**	0.065	0.084	0.025
mSR input	DPLSG	PLCC	0.068	−0.040	0.046	0.029	−0.194	−0.059	**0.081**	0.070	0.076	−0.013
		SRCC	0.022	−0.083	0.036	0.027	−0.160	−0.018	**0.077**	0.058	0.068	−0.003
	SORBD	PLCC	0.128	0.060	0.052	0.092	0.080	0.009	**0.129**	0.071	0.087	0.014
		SRCC	0.069	0.053	0.041	0.083	0.079	0.049	**0.116**	0.058	0.071	0.011
	SRIS	PLCC	0.019	0.093	0.055	0.095	0.029	0.000	**0.128**	0.060	0.065	0.007
		SRCC	0.019	0.090	0.034	0.090	0.025	0.037	**0.121**	0.041	0.061	0.011

**Table 4:** The average size proportion of salient regions over all the images.

	DPLSG		SORBD		SRIS	
	**SR**	**mSR**	**SR**	**mSR**	**SR**	**mSR**
CSIQ	0.36	0.30	0.71	0.58	0.96	0.85
LIVE	0.30	0.25	0.87	0.79	0.97	0.75
TID2013	0.30	0.24	0.79	0.68	0.98	0.82

**Table 5:** Average time cost per image on salient region detection (seconds).

	DPLSG	SORBD	SRIS
CSIQ	**3.93**	0.77	1.87
LIVE	**4.01**	0.87	2.72
TID13	**3.36**	0.66	1.41

**Table 6:** Average time cost per input on image sharpness assessment (seconds).

		MEW	CPBD	S3	FISH	SVC	LPC	MLV	SPARISH	BIBLE	RISE
CSIQ	WI	0.11	0.15	**12.03**	0.22	0.23	1.35	0.10	9.42	0.96	1.54
	CR	0.02	0.02	1.45	0.02	0.03	0.21	0.01	0.98	0.21	0.25
	DPLSG_SR	0.04	0.06	4.44	0.07	0.07	0.56	0.03	3.09	0.40	0.55
	DPLSG_mSR	0.03	0.05	3.76	0.06	0.06	0.48	0.03	2.56	0.36	0.50
	SORBD_SR	0.08	0.11	8.96	0.16	0.15	1.14	0.08	6.33	0.71	1.04
	SORBD_mSR	0.07	0.08	7.28	0.12	0.12	0.94	0.05	5.18	0.60	0.87
	SRIS_SR	0.10	0.14	11.68	0.22	0.21	1.33	0.10	9.13	0.93	1.47
	SRIS_mSR	0.09	0.13	10.34	0.18	0.18	1.27	0.09	8.08	0.83	1.28
LIVE	WI	0.15	0.20	**17.10**	0.30	0.26	1.88	0.12	13.25	1.27	1.95
	CR	0.02	0.03	2.06	0.03	0.03	0.25	0.02	1.41	0.24	0.27
	DPLSG_SR	0.05	0.06	5.43	0.09	0.08	0.65	0.04	3.81	0.46	0.62
	DPLSG_mSR	0.04	0.05	4.43	0.07	0.07	0.54	0.03	3.14	0.39	0.52
	SORBD_SR	0.12	0.16	14.67	0.26	0.21	1.90	0.10	10.69	1.10	1.60
	SORBD_mSR	0.11	0.15	13.38	0.23	0.19	1.68	0.10	9.61	1.01	1.43
	SRIS_SR	0.14	0.20	16.43	0.29	0.24	1.91	0.13	12.73	1.22	1.83
	SRIS_mSR	0.10	0.15	12.64	0.22	0.18	1.51	0.09	9.61	0.96	1.41
TID13	WI	0.07	0.12	**9.58**	0.15	0.15	1.04	0.08	7.09	0.75	1.12
	CR	0.02	0.02	1.16	0.02	0.03	0.15	0.01	0.75	0.19	0.20
	DPLSG_SR	0.03	0.04	2.88	0.05	0.05	0.37	0.02	2.10	0.31	0.37
	DPLSG_mSR	0.02	0.03	2.38	0.04	0.04	0.31	0.02	1.69	0.27	0.34
	SORBD_SR	0.06	0.08	7.48	0.12	0.11	0.96	0.06	5.31	0.61	0.87
	SORBD_mSR	0.05	0.07	6.44	0.11	0.10	0.83	0.04	4.55	0.53	0.77
	SRIS_SR	0.07	0.12	9.40	0.15	0.15	1.05	0.08	7.08	0.73	1.11
	SRIS_mSR	0.06	0.09	7.76	0.12	0.12	0.92	0.06	5.79	0.63	0.94

## Data Availability

The Gaussian blurring images used to support the findings of this study are accessible online. The CSIQ image quality assessment database is from the Oklahoma State University (https://qualinet.github.io/databases/databases/ (accessed on 5 June 2021)), the LIVE database is from the University of Texas at Austin (https://live.ece.utexas.edu/research/quality/subjective.htm (accessed on 5 June 2021)), and the TID13 database is from the Tampere University of Technology (https://qualinet.github.io/databases/image/tampere_image_database_tid2013/ (accessed on 5 June 2021)).

## Abbreviations

The following abbreviations are used in this manuscript:

HVS	human vision system
SRD	salient region detection
CNN	convolutional neural network
FCN	fully convolutional network
BISA	blind image sharpness assessment
MEW	mean edge width
CPBD	cumulative probability of blur detection
S3	spectral and spatial sharpness
FISH	fast image sharpness
SVC	singular value curve
LPC	local phase coherence
MLV	maximum local variation
SPARISH	sparse representation based image sharpness
BIBLE	blind image blur evaluation
RISE	robust image sharpness evaluation
LIVE	an image database available online
CSIQ	an image database available online
TID13	an image database available online
DPLSG	diffusion process on a 2-layer sparse graph
SORBD	saliency optimization from robust background detection
SRIS	saliency region detection and image segmentation
PLCC	Pearson linear correlation coefficient
SRCC	Spearman rank-order correlation coefficient
CR	center region
WI	whole image
SR	salient region
mSR	major salient region
